# Strengthening breast surgery workforce capacity: implementation of competency-based training programme

**DOI:** 10.3332/ecancer.2021.1203

**Published:** 2021-03-11

**Authors:** María Eugenia Aponte-Rueda, Maybell Nieves

**Affiliations:** 1 Venezuelan Breast Cancer Research and Education Foundation, Caracas 1060, Venezuela; 2 Breast Unit, Caracas University Hospital, Caracas 1040, Venezuela

**Keywords:** Breast surgery, competency-based education, implementation research, training, health workforce development, low- and middle-income countries

## Abstract

**Background and rationale:**

Quality education is a prerequisite for building a sustainable health system. To address this requirement, it is necessary to strengthen capacity and expand the training opportunities to ensure equitable and efficient development of core professional competencies for specific contexts and educational needs.

**Methods and results:**

A competency-based training programme for Breast Surgeons was built and was applied based on the Consolidated Framework for Implementation Research (CFIR). This framework provides a pragmatic structure for approaching complex interactions, multi-level and transient constructs in the real world. CFIR guided the implementation process and verified what works, where and why across each step. CFIR guided implementation was through an adaptable approach of the domains and creating relevant constructs that set up an ideal roadmap to analyse and improve learning needs, the curriculum design and the learning environment.

**Conclusion:**

The outcomes described in this manuscript demonstrate that evidence-based principles can be implemented in health professionals’ training and clinical practice even in resource-constrained settings. Building strong and sustainable healthcare workforce capacity is an urgent need for improved health service delivery and addresses real-life workplace needs in low-middle income countries. This programme integrates training with service to solve problems and develop initiatives to address existing local health priorities. While the article focuses on a training programme development, findings are shared to promote dissemination into other settings.

## Background and rationale

Breast-related diseases in all countries are an area with increasing needs, demanding a constant acquisition of knowledge due to growing scientific and technological advances in screening, diagnosis and treatments [1]. In low-middle income countries (LMICs), professional education in breast health has not been adapted to this fact, maintaining the application of inert, disjointed and outdated curricula, which produce inadequately trained breast healthcare professionals.

In these countries, the problems are systemic, with disparities between the needs of the patient and the population, poor teamwork, limited technical approaches without a local understanding, episodic orientation instead of continuous care, customarily hospital management at the expense of primary care, and weak leadership [2]. It is, therefore, vital to develop a committed, well-prepared and skilled health workforce to improve national healthcare systems’ performance, which is a prerequisite in achieving global health objectives outlined by the Millennium Development Goals [3–5]. The attention should be paid to strengthen the training and professional development by implementing functional, responsive, safe and resilient strategies [6, 7].

Hence, workforce education has witnessed a significant resurgence of broad-based health-system solutions. Quality education is a prerequisite for concrete achievement to build a robust health system [8]. To address these systemic problems mentioned above, within the national healthcare systems in LMICs, it is necessary to strengthen capacity and expand the training opportunities to ensure the development of core professional competencies for specific needs, equitably and efficiently [9] based on global knowledge. Training programmes should tailor to resource availability, support team-based learning and consider individual patient needs, preferences, values and social determinants that drive the integrated people-centred health care [10, 11], emphasising this course of action should be done with and by patients. Patients are co-creators and catalysts of needs and demands within the health system.

This process is a challenging attempt requiring the initial commitment to change and ongoing efforts tied to specific educational goals and new instructional and institutional strategies to positively impact health outcomes. As Sterman stated: ‘What prevents us from overcoming policy resistance is not a lack of resources, technical knowledge or a genuine commitment to change. What thwarts us is our lack of a meaningful systems thinking capability’ [12]. System improvements will need creativity and determination to face realities: resources-constraints and health personnel shortage due to the economic crisis and political instability.

Endeavours are most likely to succeed when they are part of innovative reform. Still, innovation cannot thrive without transformative learning and educational research that identifies the most effective breast surgery teaching patterns. Consequently, a training model should find a way to develop trainees adequately competent, i.e., making sound decisions and producing enlightened changes [13].

There is currently a call for substantial reform in medical education that is tackling tough odds, and when the context is low-middle countries, the complexity exponentially increases. In these settings, the design of educational programmes should include establishing competencies, which refer to the educational system and the health care system, considering the resource availability and population-based needs for answering the asymmetries. In a competency-based approach, a health professional’s main attribute must be the context in which she or he operates [14]. It deemphasises time-based training and compromises greater accountability, flexibility and learner-centredness [15].

Competency-based education focuses on a highly individualised learning process rather than the traditional ‘one-size-fits-all’. It simultaneously allows educational improvement, fighting against the system’s natural resistance to change, often known as the ‘not invent here’ syndrome [16]. Additionally, this competency-based education offers the opportunity to eradicate the fragmented, outdated and static system that produces a low-skilled workforce, weak in inter-professional and trans-professional skills and that lack necessary leadership to resolve ‘real life’ problems of the healthcare system.

Even though many breast surgery training programmes in all regions have launched as capacity-building initiatives, limited evidence is available about the implementation and effectiveness of such practices in LMICs [17, 18]. Therefore, this article aims to share our experience on designing and implementing a work-based training model designed to build and strengthen breast surgeons’ competencies, improve healthcare provision and meet patients and the institution’s needs adequately and efficiently.

## Beginning of academic breast surgery in an LMIC

The traditional training model of postgraduate surgical education can be described as a ‘time-spent’ model. Under supervision, they are expected to work on real-world clinical cases with the larger goal of becoming competent over this defined period [19, 20]. But, in resources-constrained settings, the main challenge involves ensuring that the trainees get sufficient exposure to procedures that they would perform in their future career, despite the given deficiencies in equipment, resources and infrastructure, which affect all aspects of providing multidisciplinary care of patients with breast diseases. This unfortunate reality poses a challenge to the programme directors; whether trainees’ experience truly ensures proficiency once the training has been completed. Hence, breast surgical training programmes in LMICs have to find ways for trainees to become adequately proficient despite the limited resources.

**Figure 1.**
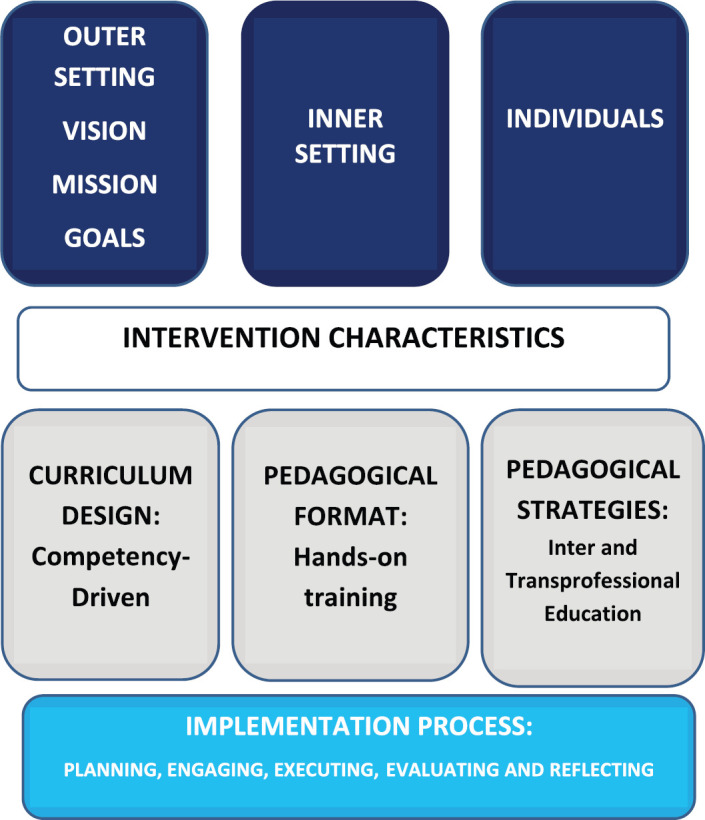
Implementation model. The five major domains of the CFIR: The intervention, the inner and outer settings, the individuals involved and the process by which the training programme is carried out.

The option is to challenge the training paradigm and consider a competency-based training model that responds to changing requirements rather than following a static programme. For that reason, we designed and developed a programme by providing tailored education, acknowledging context, practice scope and resource constraints. The training programme was implemented through the Consolidated Framework for Implementation Research (CFIR) with five domains: outer setting, intervention characteristics, inner setting, individual characteristics and process [21]. This framework provides a pragmatic structure for approaching complex interactions, multilevel and transient constructs in the real world. CFIR was used to guide an implementation theory development and verify what works, where it works and why it works across each step. Through an adaptable approach of the domains and creating relevant constructs to set up an ideal roadmap, this provided a systematic approach to analysing learning needs, curriculum design and learning environment and effectively contributes to its changes and improvements (Figure 1).

### Outer setting

Venezuela has a healthcare workforce shortage, resulting in reduced access to an already brittle healthcare system, unable to respond effectively to our current and emerging demands, such as lack of equipment, financial resources, inadequate infrastructure and referral systems. This vicious cycle is perpetuated by the limited ability to teach and produce enough medical graduates’ workforces, which has enormous breast health implications in our population [22]. Therefore, there is an urgent call to enhance in-country health workforce capacity and improve overall performance associated with responding to breast diseases. The WHO Global Health Workforce Alliance task force recommendations on education and training include strategies to expand teaching capacity by integrating practice with service, thereby maximising its impact through regional approaches [23, 24].

The outer setting includes the programme’s vision, mission and goals, which should be compatible and aligned with the institution. These serve as the basis for the development of the curriculum.

#### Vision

Building breast surgery workforce capacity through a competency-based training programme to enhance breast surgical education, improve academic-clinical knowledge, patient care, multidisciplinary approach and solve problems in a locally sensitive manner.

#### Mission

A well-prepared and pedagogically adequate breast surgeon is fundamental for developing the healthcare system in LMICs. Therefore, quality design with innovative implementation strategies for breast surgery programmes is a condition for better health outcomes.

#### Goals

- Address the breast surgeon’s role by adapting core professional competencies to local interests, according to the institution’s mission.
- Define the essential elements of the training programme, alongside the mechanisms of audit and evaluation.
- Participate in breast health programmes in tertiary hospital centres and community-based programmes. Surgical care begins in the community.
- Endorse a collaborative surgical ecosystem and non-hierarchical relationships within teams and departments.
- Understand the cross-cutting role of ethical deliberation and notions of social justice in decision-making.
- Support leadership attributes to the development of change agents, competent healthcare managers and promoters of evidence-based policies.
- Recognise the crucial role of research as an essential endeavour to mobilise scientific knowledge that originates social transformation.
- Establish rational and strategic decision-making based on evidence. Implement functional population-based interventions.
- Generate value and improve care through the strategic application of data analytics.

### Intervention characteristics

The competency-based breast surgery training programme was designed to acknowledge the local context and address the linkage between the education system demands and the health care system’s real-time needs. Education adviser and academic programme director co-produced instructional design strategies to prioritise the educational outcomes deemed necessary to train a breast surgeon who navigates and copes with the challenges faced in resource-constraint environments. This cooperative strategy provided greater accountability, flexibility and learner-centredness and the necessary balance between the content of education and community needs. Training’s core components were framed as Multidisciplinary Care, Surgical Management, Community Outreach and Screening, Patient Advocacy, Leadership and Research [25].

They focused on inter-professional and trans-professional education by recognising the importance of teamwork within the professional practice and integrating a multidisciplinary approach to managing breast diseases. They also emphasised replacing the episodic sequential treatment with cross-functional integrated care among professional and non-professional healthcare workers. Training encompasses networks between tertiary and primary care settings, providing an equilibrate environment for education by expanding academic centres to academy systems by engaging breast surgeons with local communities to navigate and handle the challenges effectively. In LMICs, surgeons are the primary healthcare providers of oncological patients, which favours continuity of care by understanding what matters to them, working by and for them (advocating for the quality of care) and coordinating and supporting their entire pathway (patient navigation process) throughout the integration of multidisciplinary approach, i.e., replacing the episodic sequential treatment by integrated care.

Similarly, the programme must build research infrastructure and provide the required data to identify solutions, i.e., allow for proactive translation of knowledge into evidence for practice and policy. The development of sound evidence-based cancer control strategies allows disseminating management and treatment options that are locally tailored. The WHO states that the ability to perform health research is a crucial factor in public health systems [26]. Clinical research should generate protocol-driven care suitable for the population and resource level [27, 28]. For this reason, the generation of research capacity is a strategic factor in the programme setting.

## Curriculum design

The process began by identifying the competencies required for addressing available resources, local practices and social determinants of health.

A search identified published work and commissioned papers relevant to competency-based surgical education [29–34]. For the concise articulation of the extensive evidence, the mixed-method strategies were used to select and prioritise the competencies. Quantitative synthesis and qualitative thematic analysis sustained the decision-making process of the taxonomy and behaviours embodied in each competency and thereby outlining the technical and non-technical skills that a breast surgery training programme should teach and assess. Functioning criteria of roles or domains of the competencies were structured to ensure patient safety throughout the training programme. The competencies were: Clinical Knowledge, Patient Care, Interpersonal and Communication Skills, Practice-Based Learning, Professionalism, System-Based Learning, Problem-Based Learning, Values and Performance (Table 1).

Finally, once the competencies were outlined, it proceeded to: defining the graduate profile, learning objectives and pedagogical strategies; selecting the clinical format; deciding on the set of targeted knowledge, skills and attitudes that capture programme goals and developing an evaluation tool that assesses trainee performances (Table 2).

## Pedagogical format

This approach focuses on hands-on training. Trainees work closely with an academic mentor within the work-based programme, allowing for a highly participatory process to address institutional breast disease patients’ burdens [35]. Under the supervised support model, the fellows have day-to-day contact with their respective mentors [36]. Mentors promote and enhance trainees’ development and fully integrate them into the institution. They build trainees’ capacity into the programme through professional advice, critical feedback and guidance while simultaneously emphasising the acquisition of non-technical skills to maximise clinical outcomes [37]. The mentor is always available to assist the trainees, either locally or remotely. The setting dictates the learning process. Work is guided by the nature of the learning process, ensuring exposure to disease management, solving breast health issues and integrating evidence-based clinical practice principles.

## Pedagogical strategies

The breast surgery training programme consists of 1 year of continuous education and work-based training, with five subjects: breast outpatient clinics, operating room, multidisciplinary meetings, rotations and research. Clinical experience at an outpatient clinic is scheduled 2 days per week, including initial outpatient assessment, preoperative decision-making, perioperative management and patient follow-up. Surgical management of breast diseases is required through operating lists. Before going into the operating room or performing breast procedures, the mentors pre-brief the trainees during the case observation and assisting as requested/required while discussing issues from a technical and non-technical perspective. After reviewing the case, there should be time set aside for a trainee debrief by giving feedback in a structured manner, which allows for continuous learning and technical improvement.

Rotations during the whole year on subspeciality services such as breast radiology, pathology, medical oncology, radiation oncology and plastic and reconstructive surgery allow the breakdown of professional silos by updating the traditional surgical model episodic and sequential treatment. In this manner, a comprehensive approach is established while collaborative relationships and team-based education are enhanced. To reinforce this understanding of integrating specialities in patient management, the trainees must participate in the weekly Breast Unit’s multidisciplinary conference.

The scholarly activity develops through a weekly scheduled educational programme: faculty lectures and journal club (with articles on interest topics). The topic selection, review and critical analysis are conducted along with the mentors and presented in a specific format. The fellows have a case-presentation and a discussion on treatment and management at the multidisciplinary case meetings. All trainees must use a web-based operative logbook to document each trainee’s academic career and list all the diagnostic and therapeutic procedures completed. The fellow’s responsibility is to ensure completion of the required number of assignments at the end and record the results. We established a logbook to track trainees’ activities. The programme director should regularly review each trainee’s operative logbook to address and identify strengths and weaknesses and support skills development. Although balancing research responsibilities with the heavy clinical load has been challenging, presentations at national meetings and publications in a peer-review journal are still expected. Research activity should be based on protocol-driven implementation science to build local research infrastructure on quality improvement projects.

### Inner setting: learning environment

Breast surgery training programme is carried out at the Caracas University Hospital. The University Hospital is an academic teaching hospital of the Central University of Venezuela that serves as a resource for the Venezuelan health care network. This institution is the main referral hospital that provides tertiary care of referred patients from peripheral regions of the country and supports local primary care centres. The programme is affiliated to and governed by the Breast Unit. The Breast Unit is included within the administrative organisation chart of the Hospital.

Breast trainees’ clinical responsibilities are dictated by the guidelines of governing resident review bodies of the Caracas University Hospital. All graduate medical education-approved training programmes are financed and accredited by the National Minister of Health in this institution. The applicant must hold a certified license as a general surgeon. Criteria for admission are linked to the training purposes; thus, a merit-based admissions policy is required to obtain a balanced composition reflective of the programme’s intention.

Breast Unit staff members integrate the faculty, ensuring that trainees will be given adequate opportunities to interact with clinicians in companion breast specialties (radiology, pathology, medical oncology, radiation oncology, and plastic and reconstructive surgery). Faculty roles and administrative structure responsibilities were defined; this included identifying the Programme Coordinator, who executes leadership and administrative tasks, carrying out the curricular issues, admission criteria, trainees’ assessment and the assignment of the research expectations. Importantly, this person serves under the supervision line of the programme director.

### Individuals

The trainee is the primary focus of the framework; the programme prioritises and articulates the competencies needed to prepare them through their career path, to empower, engage, motivate, inspire, transform and develop new leaders for the profession by the promotion of identity, adoption of commitments and the disposition to address health needs. All breast surgeons must be educated to mobilise knowledge and engage in critical reasoning with situational awareness. They must be proficient in participating in population-centred health systems. Through service-oriented activities, each trainee collaborates with each academic faculty member, steered by the ethical commitment and social accountability [38].

The training sets out an assessment strategy that is carried out throughout the year. Assessment is formative but also summative. Formative assessment is the skill, knowledge, attitudinal mentorship and performance feedback evaluated by each faculty member during the trainee’s learning process. Ratings are directly related to observed behaviours (poor –considerable improvement is needed – and acceptable – satisfactory standard). Summative assessment consists of a written and an oral part examination allowing graduation and institutional certification. The oral examination entails the presentation of a clinical research paper.

### Implementation process: practice conceptualisation

The programme aims to improve the workforce capacity using an implementation framework that allows for its adoption and adaptation in a complex environment [39, 40]. Implementation embraced the intervention potential to ensure that it was translated into evidence-based policy, and practice, by understanding real-life constructs and setting dynamics in limited-resource settings. The process comprised an iterative sequence of steps, beginning with planning and concluding with an analysis of the reflections. At all stages of the process, insights from key areas of growth were obtained, important surgical care advances were achieved and further needs were identified.

**Planning** the training began with identifying the vision, mission, goals and purposes, then designing the intervention, defining the pedagogical format and strategies, success metrics and concluding with developing an implementation plan into the learning environment.

Key areas of growth: The curriculum design and development had a dynamic approach between a pedagogical expert and an academic programme director. A coordinated approach is necessary for scale-up breast health services. The programme contemplates the acquisition and application of knowledge and skills to sustain the local capacity to improve access to safe and affordable surgical care by leveraging the existing in-country healthcare system and infrastructure. The host institution established standards for structure and procedure: a. stewardship and governance; b. normative and policy; c. definitions evidence-based for decision-making and d. implementation process. An implementation framework was employed as an iterative and action-oriented structure, notwithstanding with LMIC’s, where these interventions are underused [41].

Surgical care advances: For breast patients to access surgical care in a timely and safe manner, they need a delivery system that works. Considering that surgical care begins in local communities [42], primary care training was integrated into the programme, and the trainees’ interaction with community health workers enhanced the procedure. The idea is to support breast surgeons in practicing the breast patient care continuum and adding a primary care outpatient consult to the consult chart. Primary care outpatient consult provides early diagnostic on patients, operates as a hub for breast cancer patients’ surgical care to tertiary level and delivers a post-discharge follow-up. This network approach (primary-tertiary level) has allowed us to reduce care delays [43] by skipping referrals. Likewise, the programme seeks to avoid patients and caregivers having to navigate their means for appropriate cancer care haphazardly. The trainee follows patients throughout the entire process since screening or symptoms onset through operative care until their postoperative follow-up. The whole navigation process for patients and caregivers (who are often illiterate) is multifaceted and complex. To guide and support each step, we use smartphones as mobile-health tools to assess images, biopsy reports and postoperative surgical wounds.

Identified needs: The host institution is a national hub for healthcare; therefore, primary care outpatient consults provide a large patient volume for the trainees. Further, much care is given with limited resources, thereby creating a need to develop and implement triage guidelines and algorithms that allow quality care to rapidly and accurately identify high-acuity patients. To improve the clarity of the programme, the programme faculty should create handbooks and procedure manuals. Educational materials as clinical decision aid tools must be translated into Spanish to be locally adopted.

**Engaging** adequate staff is required to support and educate trainees by consistent clinical and academic attendance year-round. The interaction of surgical trainees with the surgical ecosystem allows them to acquire various competencies through a multidisciplinary and transdisciplinary approach.

Key areas of growth: The academic institution where the training programme develops must meet basic standards accreditation for a Breast Unit to organise breast care [44, 45]. Faculty must have a group of medical specialists dedicated to trainees’ formation. They also have to be willing to train and work with the trainees for a whole year of rotations. Hence, the unit staff must become faculty members. The faculty actively participates in the programme’s implementation; this active engagement amplifies learning achievements and enhances its acceptability. For assuring comprehensive exposure to breast diseases, external rotations complement the programme well. Further, the trainees are incorporated into local Non-governmental organisations’ activities.

Surgical care advances: Multidisciplinary rotations and task-oriented training curriculum help trainees to perform functions, solve problems and achieve multifaceted skills associated with multidisciplinary breast care. This approach allows assurance of innovative ways to resolve healthcare workers’ shortages in some areas like medical oncology. Trainees work in all rotations with breast specialists during consultations, where well-defined clinical tasks are distributed to guarantee the integral treatment of breast patients. Alliances with the private sector allow exposing trainees to cutting-edge imaging technology. A mix between public and private providers drives forward training scale-up.

Identified needs: Collaborations also have to include multi-stakeholder partnerships to ensure the intervention’s quality and sustainability, comprising local leaders backed by international collaboration.

**Executing** the intervention by moving away from the traditional in-service practice and replacing it by creating a dynamic learning environment that encourages critical thinking.

Key areas of growth: Empower trainees to customise best practices to the given context to achieve sustainability and social accountability. Social accountability is a term used by WHO as ‘*directing education, research and service activities towards addressing priority health concerns of the community, region, and nation they have the service* mandate’ [38]. In this way, the programme’s clinical practices are aligned with social health goals and, therefore, must accomplish community needs. Based on our local context, trainees’ success is supported by employing pedagogical principles that focus on knowledge translation to practice and integrate problem-solving across the care spectrum.

Surgical care advances: At the breast outpatient clinic, the trainees have to achieve prompt assessments and accurate diagnoses. Clinical examination, mammography and ultrasound are often the available (and effective) imaging resources for diagnosing a patient. Clinical and imaging correlation defines diagnostic workup, usually during the same clinic visit, as a strategy to compensate for the lack of radiologists on-site. Breast images are reviewed at weekly radiology consult with breast imaging specialist. Diagnostic needle interventions can be performed on-site, avoiding the high threshold needed to reach referral services and diminishing histological diagnosis turnaround time. In a weekly multidisciplinary meeting, all cases, i.e., benign and malignant, are reviewed and discussed to ensure accurate clinical-imaging and pathological correlation.

Identified needs: The evolving utilisation of ultrasound by breast surgeons in clinical practice should enhance our provision of care for breast disease women at resource constraint settings [46]. Breast surgeons must acquire comprehensive proficiency in ultrasound techniques and ultrasound-guided breast procedures by developing a structured breast ultrasound syllabus with local accreditation.

**Evaluating** the educational programme. The first cohort started in 2014. The main areas of impact have been: capacity building through the breast health education programme, research in locally applicable projects to highlight current care’s effectiveness and build up the continuum of medical care delivery system, addressing institutional health priorities.

Key areas of growth: The programme has survived changes in the way we worked. Resources-constraints and advanced presentation have determined us to redefine the service scope, being mindful that we had to face a shifting socio-economic context. Currently, action-oriented solutions have been tailored to resource-constraints that drive surgical decision-making and treatment [47].

Surgical care advances: Partnership with the private sector has allowed enhancing the training programme’s educational experience by increasing exposure to the whole surgical procedures spectrum and innovating technologies into breast subspecialities, as observed in the case of radiology and radiation oncology. The training faces increased rates of radical mastectomies in the Host Institution with a decrease of breast-conserving surgery, either due to a late presentation or due to the patient’s inaccessibility to receive radiotherapy or other adjuvant treatment.

It is necessary to evaluate, manage and comprehensively treat breast health problems due to breast patient’s attendance at the institution should guarantee care. Insufficient managerial support due to resource-constraint leads to poor functionality and organisation in all delivery care systems, specifically at the operating room. To fulfil this gap, trainees have to assume managerial tasks on the organisation that overwhelming them with their clinical responsibilities but added valuable benefits to the functionality of surgical care and, in particular, to planned or elective surgery.

Identified needs: Priority items specified for further work included reliable systematic data collection. Proposed short-term solutions were an electronic logbook, which is fulfilled by each student each year. This logbook works as a context-appropriate information system that allows the trainees’ metrics to be tracked and their performance reviewed. Implementation of electronic medical records is supported and needed to monitor outcomes and assessments of care. The programme maintains a database of ongoing research efforts and potential projects.

**Reflecting** this step closes the cycle by integrating the information generated through the entire process. Resource-limited settings can carry out educational and clinical best practices that enhance breast surgical education (breadth and quality). Despite improvements in the care delivery system, the workforce shortages at the hospital level, among other factors, also depend on the National Ministry of Health’s ability to retain staff in the public system.

Key areas of growth: This programme is grounded in our ongoing experiences to devise and implement training interventions that promote global equity for breast health. Sharing the learned knowledge involves meaningfully informing the implementation process, considering the context in which it is embedded, highlighting the complex and challenging problems faced in adopting and integrating evidence-based health interventions to improve local practice patterns.

Surgical care advances: The key to the process has been its ongoing nature, which has combined the analysis of results with lessons learned:

Conduct rational and strategic interventions in collaboration with experts in the field of education.

Apply a conceptual framework for capacity-building, monitoring and evaluating the intervention, integrating quality improvements.

Incorporate the programme into an existing structure using its available resources and infrastructure. To achieve an expansion of the workforce is necessary for modelling the problem.

Enhance institutional capability by strengthening the proficiency of the health workforce through in-service and real-life experiences.

Include tertiary hospital centres beside networks of primary health and community-based programmes for breast health promotion and timely access to healthcare for the community [48].

Local leadership is essential for ensuring the stability and sustainability of the programme. The programme’s chief coordinator or director is recommended to have a significant presence in the daily academic environment and practice.

Faculty Board would also be responsible for curriculum design, selecting pedagogical format, strategies, the approval and development of educational resources and the performance assessment. It is fundamental to strengthen educational resources by the faculty, such as syllabi, didactic materials, guidelines and algorithms, to allow for affordable, safe and quality care.

Identified needs:

Allow innovative and collaborative mechanisms with all sources: public, private and Non-Governmental Organisations (NGOs) for countering constraints that impede the programme’s capacity to scale-up.

Set up strategies to collect quantitative and qualitative data and their analyses to understand the local burden of disease and evaluate current care effectiveness. Quality statements can also be used to derive causal relationship maps [49].

Share generated knowledge from the design phase to the implementation process by capturing analytical reflections of what it is doing (in the context of how it is embedded) to adopt/adapt them across settings. A global perspective of best practices and best principles can generate a full understanding of health education in LMICs [50].

## Discussion

There is a significant lack of adequate public health infrastructure and approaches for tackling effective breast healthcare delivery in LMICs. Although the reasons for limited access to adequate care are complex and multifactorial, the most significant barrier is the shortage of trained breast surgical providers [51]. This shortage becomes a limitation for enlarging surgical care and addressing local health needs [52].

The WHO addresses reducing inequalities by building up adequate capacity, a process by which human and institutional resources are developed, allowing them to ‘perform functions, create solutions and achieve objectives’, [28] to provide essential and quality health services. Sustainable improvements in workforce capacity and breast health outcomes require building integrated, locally sustainable training responsible for achieving the community’s specific needs. This process is challenging, requiring the initial traditional instructional approach to change and enhancing the ongoing effort toward integrating academic-clinical teaching along with participatory learning. For accomplishing a transformative scaling up health professional education, a clear training framework is imperative that ensures effective health service.

Designing a training model in countries with financial constraints, inadequate infrastructure or push-pull factors to leave represents a comprehensive re-engineering effort [53]. In this environment, there is no alternative than to shift to a new model that includes a more intentional design process in planning the curriculum (content that is taught), using a variety of instructional methods (how it is taught) and of assessment (how the effectiveness of the educational process is measured). The goals have to be identified across the academic-clinical domains, processes, systems and techniques useful in achieving ‘best practices’. In surgical education, best practices mean understanding what happens in the teaching environment to achieve high-quality educational outcomes, which provide immense support for the surgical training experience across countries [16].

Therefore, all breast surgeons in any country must be instructed to share knowledge and ethically engage in critical thinking. They have to be competent and participate in people-centred care, as locally responsive team members should be connected globally. It is not enough to have an adequate health workforce; they must respond locally to existing health challenges and act as the first line of individual health security [54]. This requires moving outside traditional pre-service/in-service silos and creating dynamic learning environments through inter- and trans-professional education [55]. This can be achieved by establishing planning mechanisms to setting patient priorities, designing their treatment management and tracking their surveillance.

Challenging the training models requires addressing the status quo and not feeling constrained by resource scarcity. Besides, creating a culture of academic/clinical excellence that achieves aspirational goals and identifies solutions would prove to be the key to this programme’s success. Our case study provides a systemic approach for creating curriculum design based on local health and learning needs, strengthened by its implementation process, and an assessment that identifies solutions. Modifications to this training programme like the competency-based approach, redefining breast healthcare providers’ roles and integrating them into the health care system have demonstrated that it can improve the continuum of care. The competency-based approach understands the context in which the professional performs, providing opportunities to develop skills and knowledge necessary to navigate and manage the challenges faced by breast surgeons effectively [15]. The domains to be undertaken and competencies to be attained have to reflect the available resources accurately. This approach creates a new curriculum and maintains open communication into the institution for enhancing individual and institutional capacity by assuring a complement (rather than a burden) to the system. The implementation’s framework provided a lens to explore the process and consider future curricular updates [56] through a creative learning environment and multidisciplinary approach. However, significant challenges still exist and continue to persist. These themes and issues include (1) a continuous evaluation of organisational structure/practice and clinical rotations; (2) the development of leadership skills and empowerment in trainees; (3) a consideration of the impact of admission processes and academic readiness; (4) assessment of faculty capacity and theirs needs for continuing education; (5) incorporation of adequate educational resources locally-adapted and (6) a validation of a scoring system. The COVID-19 pandemic has disrupted and affected surgical training, and hence, the long-term impact on the training programme is still to be defined.

Scale-up needs in breast health require adequate strategies to systematically identify, develop and coordinate innovative initiatives by sharing and applying the generated knowledge. In low- and middle-income countries, training programmes require institutions that accredit and certify breast surgeons and well-established curricula focused on translating evidence-based knowledge to competency-driven practice to improve the breast care continuum within the local communities – creating educational content focused on learning outcomes. An effective clinical practice environment would be an ideal beginning for the curriculum design process.

## Conclusion

The outcomes described in this article demonstrate that evidence-based best principles can be implemented in health professionals’ training and clinical practice, even in resource-constrained settings. Building health workforce capacity is an urgent need to improve health service delivery and address real-life workplace needs in LMICs. This programme integrates training with service to solve problems and develop initiatives to address existing challenges, which are responsive to local and national health priorities. Key features of the programme emphasise learning strategies across the academic/clinical domains supported by faculty who supervise clinical instruction. Training involvement in an academic clinical setting should be sustained by the host institution, healthcare system and community.

## Conflicts of interest

The authors declare no conflicts of interest.

## Source of funding

Venezuelan Breast Cancer Research and Education Foundation.

## Figures and Tables

**Table 1.** Competencies taxonomy.

Competency	Description
Medical knowledge	Learning engagement: Understand established and evolved breast disease basic and clinical science: benign and malignant breast disease and high-risk lesion.
	Interpret information: Organise, analyse and interpret evidence. Question implementation strategies and their adoption.
	Critical thinking: Think innovatively when interpreting evidence, problem-solving and decision-making. Reframe constraints, challenge barriers and create innovative alternatives.
Performance	Apply knowledge and skills in surgical management, multidisciplinary care, community outreach and screening to support safety and quality, following good clinical practice codes and institutional protocols. Copy with constraints.
System-based learning	Design patient care: Create integrated care provided by a multidisciplinary team to replace disparities.
	Outline functional referral system locally adapted.
	Plan care, making it more accessible for the patient to navigate, thereby improving its experience.
	Quality improvement: Measure outcomes accurately. Develop a data science strategy to achieve precision medicine (by tailoring treatments) and create a learning health system (by predicting outcomes and identifying areas for improvement).
Problem-based learning	Define constraints: the local and global context. Delimit ethics, culture and literacy issues.
	Understand the context: Recognise the responsiveness of the health care system. Know existing system resources for patient care.
	Identify and solve real-world problems within defined constraints.
	Gather information: Use internal data to inform decisions and drive innovation.
Interpersonal and communication	Communicate effectively with patients and their caregivers – across disciplines, within listening, oral, written means.
	Teamwork: Create a vision, build consensus and offer active collaboration to inter- and trans-disciplinary approaches to achieve patient-centred care.
Professionalism	Coordinate efforts to jointly take professional and ethical responsibility for clinical management and behaviour. Envision and account for resource-constraints.
	Entrepreneurial attitude: Seek a ‘real-world’ understanding of context and constraints to improve patients outcomes.
	Lead patient care and providing management direction, demonstrating high standards of clinical practice and care.
	Promotion of identity: Adoption of commitments that support the trust of the public.
Values	Adherence to ethical and cultural principles. Commitment to professional behaviour, identity and responsibilities.
	Join in public reasoning as an informed citizen to promote enlightened transformation in the population.
	Respect for the dignity of those they serve.
Patient care	Patient-centred care: Focus on patient inputs, those that matter to them. Patients are welcome to co-create their care to the extent that they wish.
	Make sound decisions informed by context with and by patients about designing solutions and problem-solving.
	Social accountability.
	Use of information and communication technologies to enhance the learning experience for trainees.
Practice-based learning	Evidence-based practice: Appraisal of scientific evidence for improving patients care.
	Understand staff constraints and operating settings.
	Data analytics: Analyse statistical evidence. Get more knowledgeable and confident medical choices.
	Design solutions: Care pathway design to improve outcomes, taking into consideration patient inputs. Support for shared decision-making.
	Expand skills to look beyond healthcare systems to health in the community.

**Table 2.** Subjects of breast surgery training programme. Core components.

Subject	Learning objectives	Pedagogical strategies	Functioning criteria	Assessment
Breast clinic outpatient clinic for breast screening, as well as diagnostic and follow-up of breast disease patients.	To comprehend all aspects of breast diseases and provide comprehensive management. To evaluate the indications for and demonstrate proficiency in the performance and interpretation of common in-office procedures. To promote the best standard of resource-stratified breast care and screening methods. To identify and advise patients at risk of developing breast cancer. To demonstrate proficiency in pre-surgical evaluation, treatment planning, perioperative management and postoperative follow-up.	Two days per week, attending together with a specialist breast surgeon who advises, guides and supports them in the patient’s diagnostic workup, management and treatment.	Assess history and clinical symptoms and signs of benign and malignant breast disease as well as risk lesions. Apply evidence from clinical studies and guidelines in clinical work. Select, recommend and interpret imaging examinations of the breast. Performance of breast needle biopsies and interpret the results. Understand patients’ cultural environment and socio-economic disparities to explain their treatment options and facilitate their decision-making process. Identify resources available for care testing and advise patients regarding its indications. Communication with/and education to the community. Communication and interaction with cancer support groups.	Electronic recordkeeping (Logbook) to document and record the trainee’s clinical experiences, with regular progress reviews. Assessment of competences and performance feedback in the outpatient clinic. Evaluations from nurses and staff.
Operating room	To understand the surgical anatomy of the breast and axilla. To select, recommend and perform breast surgical techniques to improve cosmetic outcomes, minimise trauma and attain the best surgical outcome in major breast procedures and breast biopsies.	Operating lists per week to breast surgery, by attending together with a specialist breast surgeon, whether as the surgeon or assistant. Breast specialists pre-brief the trainees, and during the case, they observe and provide assistance to trainees as requested/required from a technical and non-technical perspective. In the end, the trainee debriefs them in a structured manner.	Perform surgical treatment with an understanding of the breast’s surgical anatomy. In-depth knowledge of indications for surgical techniques to optimise the cosmetic outcome, minimise trauma, and achieve the best oncological outcome. Management of pre-, peri- and postoperative follow-up.	Electronic recordkeeping (Logbook) to document and record trainee operative experiences, with regular reviews of progress. Assessment of competences and performance feedback in the operating room. Evaluations from nurses and staff.
Multidisciplinary team meeting	To have a handle on the multidisciplinary approach of breast patient care. To recognise the importance of interdisciplinary assessment in pre-and post-surgical treatment planning.	Trainees must attend and participate in multidisciplinary meetings once per week. Trainees must present and discuss each case – present relevant literature by choosing and reviewing it previously with mentors and presenting it in a specific format.	Cases presentation: Design of the decision-making process. Adequate preparation of educational material for other fellows, residents, students and lay audience. Evaluate literature critically.	Assessment of competences and performance feedback in cases presentation. Performance /participation in selected readings and literature review.
Rotations	To coordinate interdisciplinary management of breast care. To understand the integration of oncologic specialities and palliative care in the treatment of breast cancer patients.	Regular rotations to interact with breast companion speciality services (radiology, pathology, radiation oncology and medical oncology) and provide depth understanding and exposure to each discipline’s principles and practice.	Understand the indications (including Breast Imaging and Reporting Data System nomenclature) for and limits of breast diagnostic imaging in different age groups. Acquirement of depth knowledge of: Breast pathology benign and malignant. The use of chemotherapy, hormone therapy, biological agents, palliative care and radiotherapy for breast cancer.	Faculty summative evaluations. Assessment of competences and performance feedback for each rotation.
Research	To generate value and improve care through the strategic application of data and analytics. To provide the required data to identify solutions. To develop sound evidence-based interventions locally tailored. To generate protocol-driven care.	Participation in or observation of clinical and primary care-based research.	Collect and review a database. Design of clinical protocols Manuscript preparation and submission. Attend meetings on breast diseases.	Assessment of competences: mentorship and performance feedback. Oral presentation of a research project.
